# Effects of crocin supplementation during *in vitro* culture of intact and half-destroyed four-cell mouse embryos

**DOI:** 10.5935/1518-0557.20230050

**Published:** 2023

**Authors:** Duangrudee Peetinarak, Waraporn Piromlertamorn, Teraporn Vutyavanich, Usanee Sanmee

**Affiliations:** 1 Department of Obstetrics and Gynecology, Rajavithi hospital, Bangkok, Thailand; 2 Division of Reproductive Medicine, Department of Obstetrics and Gynecology, Chiang Mai University, Chiang Mai, Thailand

**Keywords:** crocin, blastocyst development, differential staining

## Abstract

**Objective:**

We evaluated the effects of crocin supplementation during culture of intact and half-destroyed four-cell mouse embryos. Outcomes measured included rate of cleavage arrest, blastocyst formation, and blastocyst cell number.

**Methods:**

We used laser to create two zonal holes without blastomere destruction in Groups 1 (n=100) and 2 (n=100), and to destroy two of the four blastomeres in Groups 3 (n=150) and 4 (n=150). Embryos were cultured in groups of ten in drops of medium without (Groups 1 and 3) or with 20 µg/ml of crocin supplementation (Groups 2 and 4).

**Results:**

Embryos in Groups 1 and 2 had no difference in the rate of cleavage arrest (6.0% vs. 7.0%, respectively; *p*=0.774) or blastocyst formation (89.0% *vs*. 86.0%, respectively; *p*=0.521). Neither was there a difference in the number of cells in the blastocysts (99.6±23.5 *vs*. 95.6± 8.2, respectively, *p*=0.83). Half-destroyed embryos cultured in crocin-supplemented medium (Group 4) had a lower rate of cleavage arrest (14.7% *vs*. 30.0%, *p*=0.001), and a higher rate of blastocyst formation (51.3% *vs*. 37.3%, *p*=0.015), than those in non-supplemented medium (Group 3). In blastocysts derived from half-destroyed embryos, there was no difference in the number of cells in ICM (14.5±3.9 *vs*. 13.7±2.9, *p*=0.285), TE (45.2±12.3 *vs*. 46.0±13.3, *p*=0.764), or total cells (59.7±12.2 *vs*. 59.7±14.8, respectively, *p*=0.990) among the two groups.

**Conclusions:**

Crocin supplementation during in vitro development of impaired embryos improved their development, but had no effect on intact embryos.

## INTRODUCTION

Reactive oxygen species (ROS) are normally produced in small amounts to serve as signaling molecules to regulate biological and physiological processes (Dröge, 2002; Mittler, 2017), but elevated levels are toxic to cells (Mittler, 2017). Previous studies showed that high levels of ROS, encountered during in vitro culture, adversely affected fertilization (Tatemoto *et al*., 2004), embryo development (Agarwal *et al*., 2003; Guérin *et al*., 2001) and pregnancy rates (Bedaiwy *et al*., 2004). Early cleavage embryos typically respond to ROS by exiting the cell cycle and becoming dormant in a resting stage without further cell division (Held, 2010). ROS levels during in vitro culture conditions can be reduced either by decreasing oxygen tension in the environment (Thompson *et al*., 1990) or adding antioxidants to the culture medium (Salzano *et al*., 2014; Zullo *et al*., 2016). Previous studies reported increases in blastocyst formation when antioxidants, such as β-mercaptoethanol, taurine, hypotaurine, vitamin E, and vitamin C, were supplemented in the culture medium (Agarwal *et al*., 2006).

Crocin, the main components of *Crocus sativus* (saffron), is a known natural antioxidant. It scavenges ROS, especially superoxide anion, to protect cells and tissues from the damaging effects of free radicals (Yang *et al*., 2011). Many studies reported improvements in maturation and blastocyst formation rate of mouse oocytes when low concentrations of natural saffron aqueous extract were supplemented during in vitro maturation, in vitro fertilization, and in vitro culture (Tavana *et al*., 2012; Mokhber Maleki *et al*., 2014; 2016). Incubation of bovine spermatozoa with crocin also improved sperm quality by decreasing ROS concentration and lipid peroxidation (Sapanidou *et al*., 2015).

Previous studies focused on the use of crocin to protect or enhance culture conditions. In this study, we took a slightly different perspective and looked into whether crocin might improve or rescue the development of poor-quality embryos. We used half-destroyed ICR mouse four-cell embryos as a model for poor-quality embryos to explore the beneficial effects of crocin. Outbred ICR mouse was specifically chosen because of our past experience with this model, and the experimental results were broadly applicable to human embryos (Mukaida *et al*., 1998; Vutyavanich *et al*., 2009; Sanmee *et al*., 2011).

## MATERIAL AND METHODS

### Animals

The Animal Ethics Committee (AEC) of the Faculty of Medicine, Chiang Mai University, approved the use of mice in our study.

Outbred ICR (Institute of Cancer Research) mice, nine to twenty weeks old for males and four to six weeks old for females, were procured from the National Animal Institute, Mahidol University, Bangkok, Thailand. They were housed in optimized conditions in the Animal Husbandry Unit, Faculty of Medicine, Chiang Mai University, at a room temperature of 25±2°C, 60-70% humidity, and controlled 12-hour light/12-hour dark cycles, with free access to water and food. Before the experiment, they were left undisturbed for seven days to minimize the effect of stress from transportation. This study complied with the ARRIVE guidelines (Kilkenny *et al*., 2010), and was carried out in accordance with the U.K. Animals (Scientific Procedures) Act, 1986, and associated EU Directive 2010/63/ER for animal experiment, and the international guidelines on the ethical conduct in the care and use of animals for research (National Research Council, 1996).

### Collection of 2-cell embryos

Female mice were treated intraperitoneally (IP) with ten units of pregnant mare’s serum gonadotropin (PMSG; Sigma, St. Louis, USA). After 48 hours, ovulation was triggered with ten units of hCG (Pregnyl, Merck and Co., NJ, USA) IP, followed by mating with male ICR mice. The vaginal plug was checked 16-18 hours later to confirm successful mating, and two-cell embryos were flushed from the oviducts one day after mating. Embryos were cultured in microdrops of global medium (LifeGlobal, Envimed, USA) under light mineral oil (Irvine Scientific, USA) in an atmosphere of 6% CO_2_, 5% O_2_, and 89% N_2_ until they reached the four-cell stage. Only those with an intact zona pellucida, equal blastomeres, and no fragmentation were chosen for the experiments.

### Interventions

Four-cell embryos in 10 µl drops of global medium under oil were placed on a heated microscopic stage at 37°C. A non-contact infrared diode laser (Zilos-tk Laser^®^, Hamilton Thorne Research, Beverly, MA, USA; power 300 mW, wavelength 1460 nm) was used at a pulse of 500 µs, power of 75%, to damage two of the four blastomeres (half-destroyed groups, n=300) at an area that the blastomeres touched the inner side of the zona pellucida. In the intact four-cell groups (n=200), the same pulse of infrared laser was employed to create two holes in the zona away from any blastomeres. No more than five embryos were manipulated at a time.

The embryos were divided into four groups: intact four-cell embryos cultured in medium without (Group 1, n=100) or with crocin supplementation (Group 2, n=100), half-destroyed four-cell embryos in medium without (Group 3, n=150) or with crocin supplementation (Group 4, n=150). They were cultured in groups of ten embryos per 10 µl of global medium under oil at 37°C in an atmosphere of 6% CO_2_, 5% O_2_, and 89% N_2_.

The concentration of crocin (Sigma Chemical, St. Louis, MO, USA) used in this experiment (20 µg/ml) was derived from our pilot dose-finding study, which involved nine replicates of control and case groups. In the pilot study, the crocin-supplemented groups of half-destroyed four-cell embryos achieved a blastulation rate of 67.4%, while the non-supplemented group had a blastulation rate of 42.3%. Given a Type I error = 0.05 (two-tailed) and a statistical power of 80%, we estimated that a sample size of 106 four-cells embryos would be required in each study group.

### Assessment of embryo development

Embryo development was assessed daily on an inverted microscope until the blastocyst stage was reached. The blastocysts were categorized based on the morphological criteria proposed by Gardner *et al*. (2000) for human blastocysts, as follows: early blastocyst, blastocyst, full blastocyst, expanded blastocyst, hatching, and hatched blastocyst. For this study, only full + expanded + hatching/hatched blastocysts at 72 hours of culture were considered to be good-quality blastocysts.

### Assessment of blastocyst cell number

Only good-quality blastocysts were assessed by differential staining of inner cell mass (ICM) and trophectoderm (TE) using the protocol described by Pampfer *et al*. (1990), with slight modifications. Briefly, the blastocysts were immersed into 0.5% protease (Sigma P8811) for 10-15 min at 37°C to remove the zona pellucida and washed three times in calciumand magnesium-free phosphate buffered solution (PBS, Gibco, USA). They were then exposed to rabbit anti-mouse antibody (Sigma M5774) for 30 min at 37°C, washed in calciumand magnesium-free PBS, and moved into a solution containing Guinea pig complement serum (Sigma S1639) at a concentration of 1:4, 10 µg/ml of Hoechst 33342 (Sigma H1399), and 20 µg/ml of Propidium Iodide (PI, Sigma P4170) for 30 min at 37°C. The blastocysts were washed and placed on a glass slide to allow air drying. The slides were covered with coverslips and mounted with glycerol. The cell were counted on a fluorescence microscope (Nikon E600) with an excitation filter of 515-560 nm, a barrier filter of 590 nm, and analyzed in the LUCIA FISH Program (Laboratory Imaging, Czech). ICM and TE cells were visualized in blue and red, respectively, on the fluorescence microscope.

### Statistical analysis

Statistical analyses were performed on SPSS version 22. The rates of cleavage arrest and blastocyst formation between the crocin-supplemented and non-supplemented groups were compared by Chi-square tests. The average number of cells in the blastocysts (ICM, TE, Total cells, and ICM/TE ratio) were presented as mean ± SD and compared with non-paired t-tests. A *p*-value < 0.05 was considered to be statistically significant.

## RESULTS

Five hundred four-cell embryos were included in the study. The embryos were divided into four groups: intact four-cell embryos cultured in medium without (Group 1, n=100) or with crocin supplementation (Group 2, n=100), half-destroyed four-cell embryos in medium without (Group 3, n=150) or with crocin supplementation (Group 4; n=150).

In the groups of intact four-cell embryos, there was no significant difference in the rate of cleavage arrest (6.0% *vs*. 7.0%; *p*=0.774) or blastocyst formation (89.0% *vs*. 86.0%; *p*=0.521) in those cultured with or without crocin supplementation (Table 1). The half-destroyed four-cell embryos cultured in crocin-supplemented medium had a significantly lower rate of cleavage arrest (14.7% *vs*. 30.0%, *p*=0.001) and higher rate of blastocyst formation (51.3% *vs*. 37.3%, *p*=0.015) than those in non-supplemented medium (Table 1).

**Table 1:** Effects of 20 µg/ml crocin supplementation during in vitro culture on the rate of cleavage arrest and blastocyst formation of four-cell mouse embryos.

	Intact 4-Cell Embryos		*p*-value	Half-Destroyed Embryos		*p*-value
	Control (n=100)	Crocin (n=100)		Control (n=150)	Crocin (n=150)	
Cleavage arrest^*^	7 (7.0%)	6 (6.0%)	0.774	45 (30.0%)	22 (14.7%)	0.001
Good-quality Blastocyst formation^†^	86 (86.0%)	89 (89.0%)	0.521	56 (37.3%)	77 (51.3%)	0.015

* Cleavage arrest observed at 24 hours

† Good-quality blastocyst is defined as full + expanding + hatching/hatched blastocyst at 72 hours of culture

Chi-square tests.

There was no significant difference in the number of cells in the ICM, trophectoderm, or total cells among the crocin-supplemented and non-supplemented groups in both intact and halfdestroyed four-cell embryos (Table 2). The total number of cells in blastocysts derived from half-destroyed embryos were approximately 60% of those derived from intact four-cells embryos.

**Table 2:** Cell numbers in blastocysts derived from intact and half-destroyed 4-cell mouse embryos, cultured in medium with or without 20 µg/ml crocin.

	Blastocysts from Intact 4-Cell Embryos		*p*-value	Blastocysts from Half-Destroyed 4-Cell Embryos		*p*-value
	Control (n=47)	Crocin (n=45)		Control (n=36)	Crocin (n=51)	
ICM	27.9±9.7	26.7±1.4	0.887	13.7±2.9	14.5±3.9	0.285
TE	67.7±21.8	72.9±17.7	0.525	46.0±13.3	45.2±12.3	0.764
Total cells	95.6±28.2	99.6±23.5	0.831	59.7±14.8	59.7±12.2	0.990
ICM:TE Ratio	0.4±0.2	0.4±0.1	0.162	0.3±0.1	0.4±0.1	0.272

Values are presented as mean ± SD.

Non-paired t-tests

ICM = Inner cell mass, TE = trophectoderm

Some blastocysts were excluded due to accidental loss or poor staining.

## DISCUSSION

In an IVF laboratory, it is common to have a mixture of goodand poor-quality embryos in a cohort of fertilized oocytes obtained from a stimulated cycle. As mature oocytes in a stimulated cycle came from a cohort of follicles that had different sizes, intra-follicular milieu and vascularization, they could give rise to zygotes and embryos with different developmental potential. Indeed, some studies showed that there was a positive correlation between follicular diameter and embryo quality (Ectors *et al*., 1997; Bergh *et al*., 1998; Rosen *et al*., 2008). The underlying mechanism is still unknown, but cytoplasmic immaturity is postulated to play a role. Since antioxidant enzymes including glutathione peroxidase and manganese superoxide dismutase (MnSOD) are considered to be markers of cytoplasmic maturation, it is quite possible that reactive oxygen species (ROS) play a key role in determining oocyte and embryo quality (Agarwal *et al*., 2006).

In human *in vitro* fertilization (IVF), there is a high frequency of embryonic arrest and fewer than 50% of fertilized oocytes reach the blastocyst stage. In approximately half of them, cleavage arrest might be explained by chromosome abnormalities. However, the underlying causes of developmental arrest in the remaining embryos remains unclear. It has been speculated that reactive oxygen species (ROS) might be an important causative factor (Betts & Madan, 2008). If so, the use of antioxidants might be of tremendous help, since every single embryo counts, especially in aging women and poor responders.

In this study, we used mouse embryos to investigate whether crocin might rescue the developmental potential of poor-quality embryos. Although mouse embryos are often used as a model for human embryos, they differ in certain aspects. When exposed to suboptimal culture conditions, mouse embryos undergo early cleavage arrest rather than go on dividing and giving rise to fragmented embryos (Winston & Johnson, 1992). To circumvent the problems of recruiting a homogeneous cohort of fragmented mouse embryos for the study, we chose to artificially create them by delivering the same intensity of laser to inflict injuries on two of the four blastomeres. Intact four-cell embryos, with similar holes in the zona, served as comparison groups. Crocin was chosen because it is an inexpensive non-toxic plant extract that is available to us, and there have been many publications confirming its efficacy and safety.

Although all embryos survived the laser manipulation, the injured blastomeres became swollen, then shrunk and finally formed dark condensed debris adjacent to the apparently normal blastomeres by the next day. We postulated that the acute injury induced by the laser pulse was severe enough to trigger a highly regulated program of cell death, known as apoptosis. This was in sharp contrast to necrosis, which occurs when blastomeres are exposed to extreme injury that causes disruption of their plasma membrane, with the release of cytoplasmic contents including lysosomal enzymes. In our case, dark shrunken apoptotic bodies were found next to their normal-appearing blastomeres that often underwent further cleavage division. It is well known that ROS are signaling molecules that stimulate apoptosis (Covarrubias *et al*., 2008). ROS can activate the mitogen-activated protein kinase (MAPK) pathway, and the pro-apoptotic B-cell lymphoma-2 (Bcl-2) proteins that result in mitochondrial-dependent cell death (intrinsic pathway) (Covarrubias *et al*., 2008). ROS also activate cell surface death receptors, which are involved in the extrinsic (receptor-mediated) pathway of apoptosis (Covarrubias *et al*., 2008). It was, therefore, logical to postulate that one might “rescue” partially damaged embryos either by immediately removing the injured blastomeres or adding antioxidants to the culture media. The first method required a micromanipulation procedure and skills, and imposed a risk of damage to the embryos. The second option was, perhaps, easier and preferable, and was thus chosen for this study.

We found that crocin significantly reduced the rate of cleavage arrest and increased the rate of blastocyst formation. However, the end results were significantly inferior to those in the intact four-cell groups, with or without crocin-supplementation. This might be due to the fact that embryonic mass was reduced by half compared to the intact four-cell groups, and crocin could partially overcome the adverse effects of ROS. As suggested by Soeda *et al*. (2007), crocin inhibited oxidative stress through a GSH-dependent mechanism. However, the dose of crocin in this study might not have been enough to totally reverse the adverse effect of ROS generated, or other mechanisms might be involved. Moreover, the use of crocin as a single antioxidant in our study was different from the in vivo situation, where antioxidants interact with each other in a complex system that facilitates their cycling back to reduced forms (Truong *et al*., 2016). Perhaps, the use of a combination of antioxidants that could regenerate themselves might be a preferable option worth studying.

We assessed blastocyst quality based on morphology and by counting the number of cells in the inner cell mass and trophectoderm. The number of cells in the blastocysts that developed from half-destroyed four-cell embryos was the same, regardless of crocin supplementation. This number was approximately 60% of the number seen in the blastocysts derived from intact four-cell embryos. This was in contrast to the study by Truong *et al*. (2016), which reported an increase not only in the blastocyst formation rate, but also in blastocyst cell numbers. This might be explained by the difference in doses and types of antioxidants employed, and the culture conditions, such as culture medium, ambient O_2_ concentration, embryo density in the culture drops, etc.

In our study, we cultured ten mouse embryos in 10 µl of medium oil in an atmosphere of low oxygen tension. In such conditions, the embryos were probably well protected from oxidative stress, which might explain why we did not observe any significant difference in the rates of cleavage arrest or blastocyst formation in the intact four-cell groups, with or without crocin supplementation. In this study, we used a one-step culture system, without medium replacement. Questions remained whether crocin-supplemented medium should be replenished daily or every other day to supply fresh antioxidants to combat the newly generated ROS. As we did not transfer the blastocysts into pseudopregnant mice, we had no information on implantation rate, fetal development, and live birth rate, which are the most important clinical outcomes.

In conclusion, the results of this study demonstrated that crocin supplementation improved the in vitro development of impaired embryos. Antioxidants might have a role in improving embryo quality in certain cases. Further studies should be performed before this recommendation is extended to human IVF treatment.
